# Transition metal alloying effect on the phosphoric acid adsorption strength of Pt nanoparticles: an experimental and density functional theory study

**DOI:** 10.1038/s41598-017-06812-w

**Published:** 2017-08-03

**Authors:** Hee-Young Park, Dong-Hee Lim, Sung Jong Yoo, Hyoung-Juhn Kim, Dirk Henkensmeier, Jin Young Kim, Hyung Chul Ham, Jong Hyun Jang

**Affiliations:** 1 Fuel Cell Research Center, Korea Institute of Science and Technology, Hwarangno 14-gil 5, Seongbuk-gu, Seoul, 02792 Republic of Korea; 2 Department of Environmental Engineering, Chungbuk National University, Cheongju Chungbuk, 28644 Republic of Korea; 3 Division of Energy & Environment Technology, KIST School, Korea University of Science and Technology, Seoul, 02792 Republic of Korea; 4 Green School, Korea University, Seoul, 02841 Republic of Korea

## Abstract

The effect of alloying with transition metals (Ni, Co, Fe) on the adsorption strength of phosphoric acid on Pt alloy surfaces was investigated using electrochemical analysis and first-principles calculations. Cyclic voltammograms of carbon-supported Pt_3_M/C (M = Ni, Co, and Fe) electrocatalysts in 0.1 M HClO_4_ with and without 0.01 M H_3_PO_4_ revealed that the phosphoric acid adsorption charge density near the onset potential on the nanoparticle surfaces was decreased by alloying with transition metals in the order Co, Fe, Ni. First-principles calculations based on density functional theory confirmed that the adsorption strength of phosphoric acid was weakened by alloying with transition metals, in the same order as that observed in the electrochemical analysis. The simulation suggested that the weaker phosphoric acid adsorption can be attributed to a lowered density of states near the Fermi level due to alloying with transition metals.

## Introduction

High-temperature polymer electrolyte membrane fuel cells (HT-PEMFCs) that use phosphoric-acid-doped polybenzimidazole as a proton exchange membrane are a promising power supply for stationary applications such as undisrupted power supplies and combined heat and power systems. The high operating temperatures of HT-PEMFCs (150–200 °C) can provide the advantages of faster reaction kinetics, higher tolerance to CO poisoning, easier water management, and more efficient heat utilization^1^. However, the oxygen reduction reaction (ORR) in HT-PEMFCs is typically very slow and therefore requires a high Pt loading^2^ compared to that of conventional PEMFCs operated at 80 °C^3^. Pt poisoning in the presence of phosphoric acid has been reported to be responsible for the low ORR activity in HT-PEMFCs^4, 5^. Therefore, to improve the ORR activity in HT-PEMFCs, high phosphoric acid tolerance is very important, in addition to the higher intrinsic activity of electrocatalysts without phosphoric acid adsorption.

When phosphoric acid is added to electrolytes, the electrocatalyst surface will be significantly adsorbed by phosphoric acid and its anions^6^, which typically decreases the ORR activity^4, 5^. Adsorbed dihydrogen phosphate (H_2_PO_4,ad_) was found to be the major species on polycrystalline Pt electrode surfaces using Fourier transform infrared analysis in acidic conditions^6^. Undissociated phosphoric acid (H_3_PO_4,ad_) was also detected in a strong acidic (pH = 0.23) solution^6^. The H_2_PO_4,ad_ bonded with the Pt(111) surfaces through the oxygen atoms (C_2V_ symmetry). The Gibbs free energy for H_2_PO_4_^−^ adsorption was reported to be −120 to −170 kJ mol^−1^ on Pt(111) surfaces at 0.5–0.8 V with respect to the reversible hydrogen electrode (RHE)^7^. The phosphoric acid coverage reached a maximum at as low as around 0.3 V on the Pt(100) and Pt(110) surfaces^5^. The Pt(111) surfaces showed a higher potential at the maximum H_2_PO_4,ad_ coverage (around 0.7 V vs. RHE)^7^. This finding implies that under HT-PEMFC operating potentials (≥0.6 V)^5^, the Pt(100) and Pt(110) surfaces would experience the maximum phosphoric acid coverage, and only a few areas of the Pt(111) surfaces may be free from the maximum phosphoric acid coverage.

The adsorbed phosphoric acid suppresses the ORR activity of Pt by a factor of 53.7%, 65.0%, and 86.3% on the Pt(100), Pt(110), and Pt(111) surfaces, respectively, in 0.01 M H_3_PO_4_ + 0.1 M HClO_4_^5^. As in the case of ORR suppression by sulfate adsorption^8^, the decreased number of active sites due to adsorbed phosphoric acid is the most feasible explanation for the suppressed ORR activity. As the Pt surfaces are totally covered at relatively low electrode potentials, the ORR activity determined at 0.85 V may be that at the maximum phosphoric acid coverage. Interactions between the adsorbed phosphoric acid and ORR reaction intermediates may alter the ORR activity, but they have not been investigated.

In practical terms, phosphoric acid coverage on the (111) facet of a Pt electrocatalyst is considered to occur before that on the other active sites in Pt nanoparticle electrocatalysts, such as the (100) facet, edge, and corner sites. As the coverage on the Pt(111) facet increases under the more anodic potential applied as the operating potential of HT-PEMFCs (≥0.6 V)^5^, the phosphoric acid coverage of the Pt(111) facet in that potential region could be decreased by modifying the Pt(111) facet; as a result, improved phosphoric acid tolerance could be achieved. However, as the Pt(100) facet is fully covered by phosphoric acid anions at a more negative potential (around 0.3 V vs. RHE)^5^, the phosphoric acid coverage of the Pt(100) facet at 0.6 V is considered hard to change. Thus, decreasing the phosphoric acid coverage on the Pt(111) facet is considered to be crucial for realizing improved phosphoric acid tolerance. Moreover, improving the phosphoric acid tolerance of Pt(111) surface is an effective method to enhance the ORR activity of nanoparticle electrocatalysts, because of the higher ORR activity of Pt(111) surfaces as compared to the Pt(100) and Pt(110) surfaces, although the ORR of Pt(100) and edge/step sites in the nanoparticles should not be overlooked.

To decrease the phosphoric acid coverage on the Pt(111) facet, it is considered necessary to decrease the adsorption strength of phosphoric acid on the Pt(111) facet, as the equilibrium coverage at a certain potential is decreased by decreasing the adsorption strength of phosphoric acid^9^.

As phosphoric acid adsorption and oxygen (hydroxide) adsorption form similar chemical bonds, *i.e*., Pt–O, it can be assumed that the adsorption strength of phosphoric acid on Pt–transition metals alloys (PtM) is weaker than that on Pt, like the weaker OH or O adsorption on PtM than on Pt, because of the lowered d-band position of PtM surfaces^10^. However, to the best of our knowledge, there is no systematic study on the adsorption strength of phosphoric acid on PtM surfaces. He *et al*. reported that the PtNi nanoparticles showed less decreased ORR activity upon phosphoric acid addition as compared to that of the Pt nanoparticles;^4^ however, the phosphoric acid tolerance of PtNi was attributed to changes in the adsorption geometry. Only two studies have reported the adsorption strength of phosphoric acid anions on Pt(111) surfaces using a computational simulation^11^ and cyclic voltammetry (CV) analysis^7^.

Further, PtM has been widely reported to have higher intrinsic activity than pure Pt owing to the lowered adsorption strength of the reaction intermediate^10, 12–14^. Owing to PtM’s high intrinsic activity, it has been widely tested as an electrocatalyst for HT-PEMFCs using the half-cell^4, 5, 15–17^ and single-cell tests^16, 18, 19^. For example, the ORR activity of a carbon-supported Pt_3_Ni electrocatalyst (Pt_3_Ni/C) measured in purified 100% H_3_PO_4_ at 190 °C was 1.6 times higher than that of Pt/C when the activity was normalized with respect to the Pt area. However, to the best of our knowledge, whether the effect of alloying on the adsorption strength of phosphoric acid can affect the phosphoric acid tolerance has not been investigated.

In this research, we investigated the effect of alloying on the adsorption strength of phosphoric acid anions using electrochemical analysis and first-principles calculations. As the surface of alloy electrocatalysts in an acidic condition is known to have a Pt skin structure due to dissolution of transition metals, while the core of the nanoparticles maintains the alloy structure^20^, the Pt skin and alloy core structure was used in the electrochemical analysis and computational simulation. CV was used to analyze the adsorption strength of phosphoric acid anions on carbon-supported Pt_3_M alloy (M = Ni, Co, and Fe) nanoparticles (Pt_3_M/C) in order to validate the simulation results. The adsorption strength of phosphoric acid was predicted using density functional theory (DFT) calculations with model structures consisting of a Pt skin layer on Pt_3_M(111), where M = Ni, Co, and Fe. Then, the effect of alloying on the adsorption strength of phosphoric acid was discussed on the basis of the adsorption behavior of H_2_PO_4_ and electronic properties such as the d-band center and density of states near the Fermi level.

## Results and Discussion

**Figure 1:**
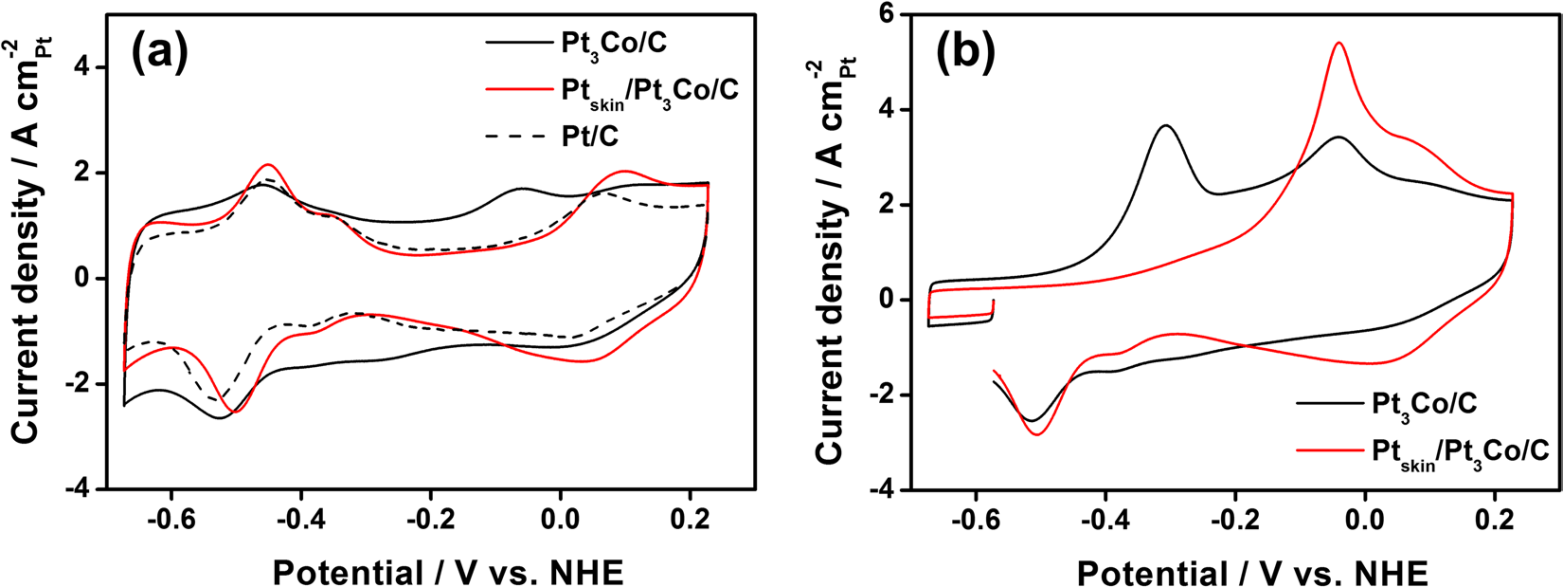
Cyclic voltammograms of the electrocatalysts. (**a**) pristine Pt_3_Co/C, Pt_skin_/Pt_3_Co/C, and Pt/C in 0.1 M KOH and (**b**) electrochemical oxidation of adsorbed CO on pristine Pt_3_Co/C and Pt_skin_/Pt_3_Co/C.

The surface structures of Pt-terminated samples (Pt_skin_/Pt_3_Co/C, Pt_skin_/Pt_3_Ni/C, and Pt_skin_/Pt_3_Fe/C) were analyzed by the electrochemical techniques of CV and CO-stripping voltammetry. In CV, the pristine Pt_3_Co/C showed a Co oxidation peak around −0.05 V^21^, in addition to the oxidation peaks for hydrogen desorption on Pt (ca. −0.45 V) and Pt oxidation (>0.1 V)^22^ (Fig. 1a). After electrochemical Co dissolution and surface Pt reduction (Pt_skin_/Pt_3_Co/C), which were realized by CV treatment at 0–1.0 V and −0.2 to 0.4 V, respectively, the Co oxidation peak (−0.05 V) disappeared, and the Pt-related signals (peak at −0.45 V and plateau at >0.1 V) increased. For Pt_skin_/Pt_3_Co/C, the Pt oxidation peak appeared at a more positive potential (0.099 V) compared to that of Pt/C (0.063 V) because of the effect of subsurface Co atoms, which weakened the oxygen adsorption on the Pt skin layer^23^. CV analysis of Pt_skin_/Pt_3_Ni/C and Pt_skin_/Pt_3_Fe/C revealed similar characteristics, confirming their Pt skin structure (Supplementary Information, Fig. S1). CO-stripping voltammetry also supported Pt skin formation in the alloy catalysts. Note that 0.1 M KOH was utilized as the electrolyte solution for CO stripping in this study, as acidic electrolytes can modify the surface composition because of transition metal dissolution. Pristine Pt_3_Co/C showed two oxidation peaks at ca. −0.3 and −0.05 V, which correspond to electrochemical oxidation of CO by adsorbed OH on surface Co and Pt atoms^24^, respectively. After the electrochemical Co dissolution and Pt reduction process, a single oxidation peak at −0.05 V (Pt) was observed with an increased peak area, whereas another peak appeared at ca. −0.3 V (Co). The results of energy dispersive X-ray spectroscopy (EDS) analysis (Figure S2) revealed that the Pt skin of Pt_skin_/Pt_3_M/C is mainly composed of monolayers, with a small proportion of multiple layers.

**Figure 2:**
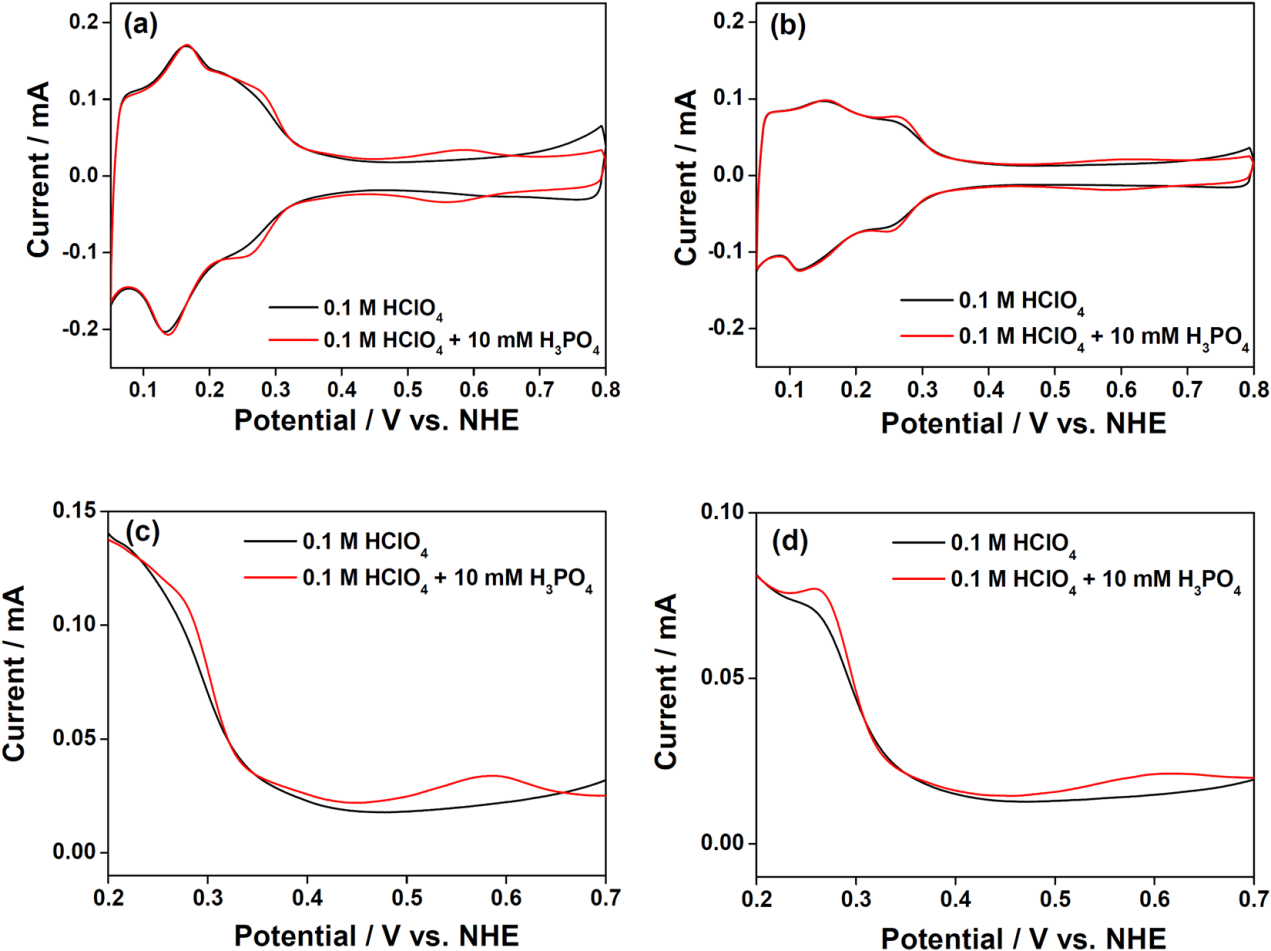
Comparison of cyclic voltammograms of the electrocatalyst with and without phosphoric acid in the electrolyte. (**a**) Pt/C and (**b**) Pt_skin_/Pt_3_Co/C electrocatalyst in 0.1 M HClO_4_ with (red) and without (black) 0.01 M H_3_PO_4_. Magnified anodic sweep of cyclic voltammograms of (**c**) Pt/C and (**d**) Pt_skin_/Pt_3_Co/C electrocatalyst.

Figure 2a shows the effect of phosphoric acid on the CV curves of Pt/C, where the anodic potential limit was fixed at 0.8 V to prevent irreversible oxidation of the Pt surfaces and the resultant surface roughening^25^. In 0.1 M HClO_4_ electrolyte, Pt/C showed characteristics typical of polycrystalline Pt surfaces: hydrogen adsorption/desorption (~0.35 V), double-layer charge/discharge (0.35–0.55 V), and Pt oxidation/reduction (~0.55 V). When H_3_PO_4_ was added (0.1 M HClO_4_ + 10 mM), new redox peaks appeared at 0.25 and 0.55 V, which correspond to electrochemical H_2_PO_4_^−^ adsorption/desorption (reaction 1) on the (100) and (111) facets of the Pt nanoparticle catalysts, respectively^5^. This indicates that the Pt(100) facet is favored over the Pt(111) facet for H_2_PO_4_^−^ adsorption.

1 $${{\rm{H}}}_{2}{{{\rm{PO}}}_{4}}^{-}\,+\,{(\text{nH}}_{2}{\rm{O}}+\text{Pt})\to (n\,-\,x{{\rm{H}}}_{2}{{\rm{O}}+{\rm{H}}}_{2}{{\rm{PO}}}_{4,\text{ad}}-\text{Pt})+x{{\rm{H}}}_{2}{{\rm{O}}+e}^{-}$$

A similar CV analysis was carried out for Pt_skin_/Pt_3_M/C to analyze the effect of alloying on the adsorption strength of phosphoric acid. On the Pt_skin_/Pt_3_Co/C electrocatalyst surfaces, phosphoric acid adsorption on the (111) facet occurred at a potential 30 mV higher than that of Pt/C, whereas the shape of the CV curve was very similar (Fig. 2b). Pt_skin_/Pt_3_Ni/C and Pt_skin_/Pt_3_Fe/C also showed a positive shift of the adsorption peak (Fig. S5). The positive shift of the phosphoric acid adsorption peak indicated weaker adsorption on the Pt_skin_/Pt_3_M/C surfaces than on that of Pt/C. However, because the phosphoric acid adsorption near the peak overlaps significantly with the Pt oxidation reaction, the adsorption strength of phosphoric acid on the Pt_skin_/Pt_3_M/C surfaces could not be investigated directly by comparing the peak position of phosphoric acid adsorption.

To evaluate the adsorption strength of phosphoric acid, the phosphoric acid coverage of the electrocatalysts near the onset potential of the adsorption was compared using the accumulated charge density of phosphoric acid adsorption from 0.35 V. As the coverage is governed by the adsorption strength of the substrate and electrode potential^11^, a higher coverage at a certain potential indicates stronger adsorption of phosphoric acid. The adsorption strength of phosphoric acid was thought to be clearly investigated near the onset potential of phosphoric adsorption (around 0.35 V) on the (111) facets because the interaction between the adsorbed phosphoric acid molecules is insignificant owing to the low coverage, and interference with phosphoric acid adsorption by Pt oxidation was negligible.

**Figure 3:**
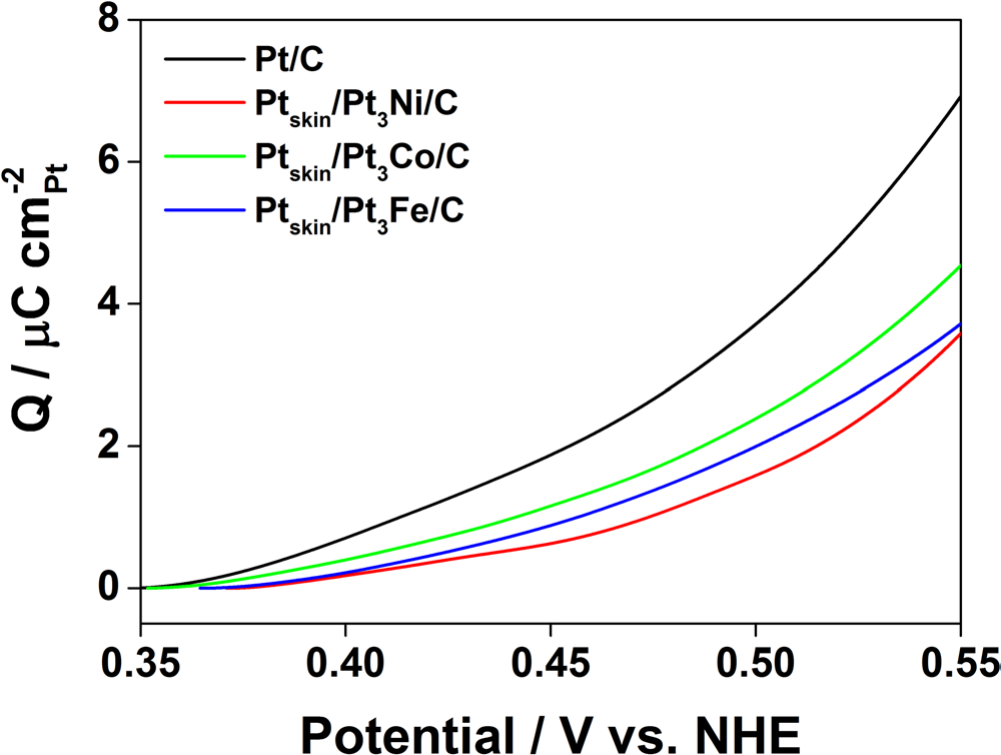
Evaluation of phosphoric acid adsorption charge density. Accumulated charge density profiles of Pt/C, Pt_skin_/Pt_3_Ni/C, Pt_skin_/Pt_3_Co/C, and Pt_skin_/Pt_3_Fe/C.

For each electrocatalyst, the current density related to phosphoric acid adsorption was determined by subtracting the current density with added phosphoric acid from that without phosphoric acid, and integrating from 0.35 V. As the underpotential adsorption/desorption of hydrogen on the Pt(111) surface was not altered by the phosphoric acid adsorption^7^, the abovementioned difference provided the current related to phosphoric acid adsorption. Figure 3 shows the accumulated charge density of phosphoric acid adsorption as a function of electrode potential. The accumulated charge density from 0.35 to 0.45 V decreased in the order Pt/C (1.982 μC cm_Pt_^−2^), Pt_skin_/Pt_3_Co/C (1.221 μC cm_Pt_^−2^), Pt_skin_/Pt_3_Fe/C (0.933 μC cm_Pt_^−2^), Pt_skin_/Pt_3_Ni/C (0.643 μC cm_Pt_^−2^). The accumulated charge density clearly indicated that the phosphoric acid anion adsorption strength showed the order Pt/C, Pt/Pt_3_Co/C, Pt/Pt_3_Fe/C, Pt/Pt_3_Ni/C.

On the basis of the experimental observations, we investigated the effect of subsurface M atoms on the adsorption strength of H_2_PO_4,ad_ on the Pt(111) surfaces by calculating the binding energies (Δ*E*_ad_) of H_2_PO_4_ on the Pt(111) and Pt_skin_/Pt_3_M surfaces for different M atoms. We selected the H_2_PO_4_ molecule to capture the phosphoric acid tolerance of the catalysts because H_2_PO_4_ is responsible for determining the adsorption strength of H_2_PO_4_^−^, as the free energy of H_2_PO_4_^−^, H_2_O_aq_, and e^−^ in the electrochemical adsorption of phosphoric acid (Eq. (1)) is independent of the adsorption site of H_2_PO_4,ad_, and the free energy differences in H_2_O_ad_ on the Pt(111) and Pt_skin_/Pt_3_M surfaces (M = Ni, Co, Fe) are reportedly as low as 0.03 eV^26^. Thus, Δ*E*_ad_ is considered to be a good descriptor for the adsorption strength of H_2_PO_4_^−^.

2 $${\rm{\Delta }}{E}_{\text{ad},X}=E({{\rm{H}}}_{2}{{\rm{PO}}}_{4,\text{ad}}-{\rm{X}})-E({\rm{X}})-E({{\rm{H}}}_{2}{{\rm{PO}}}_{4}),$$

where *E*(H_2_PO_4,ad_ − X), *E*(X), and *E*(H_2_PO_4,ad_) are the total energies of the X surfaces (X = Pt(111) and Pt_skin_/Pt_3_M) with adsorbed H_2_PO_4,ad_, bare X surfaces, and a gas-phase molecule of H_2_PO_4,gas_, respectively.

**Figure 4:**
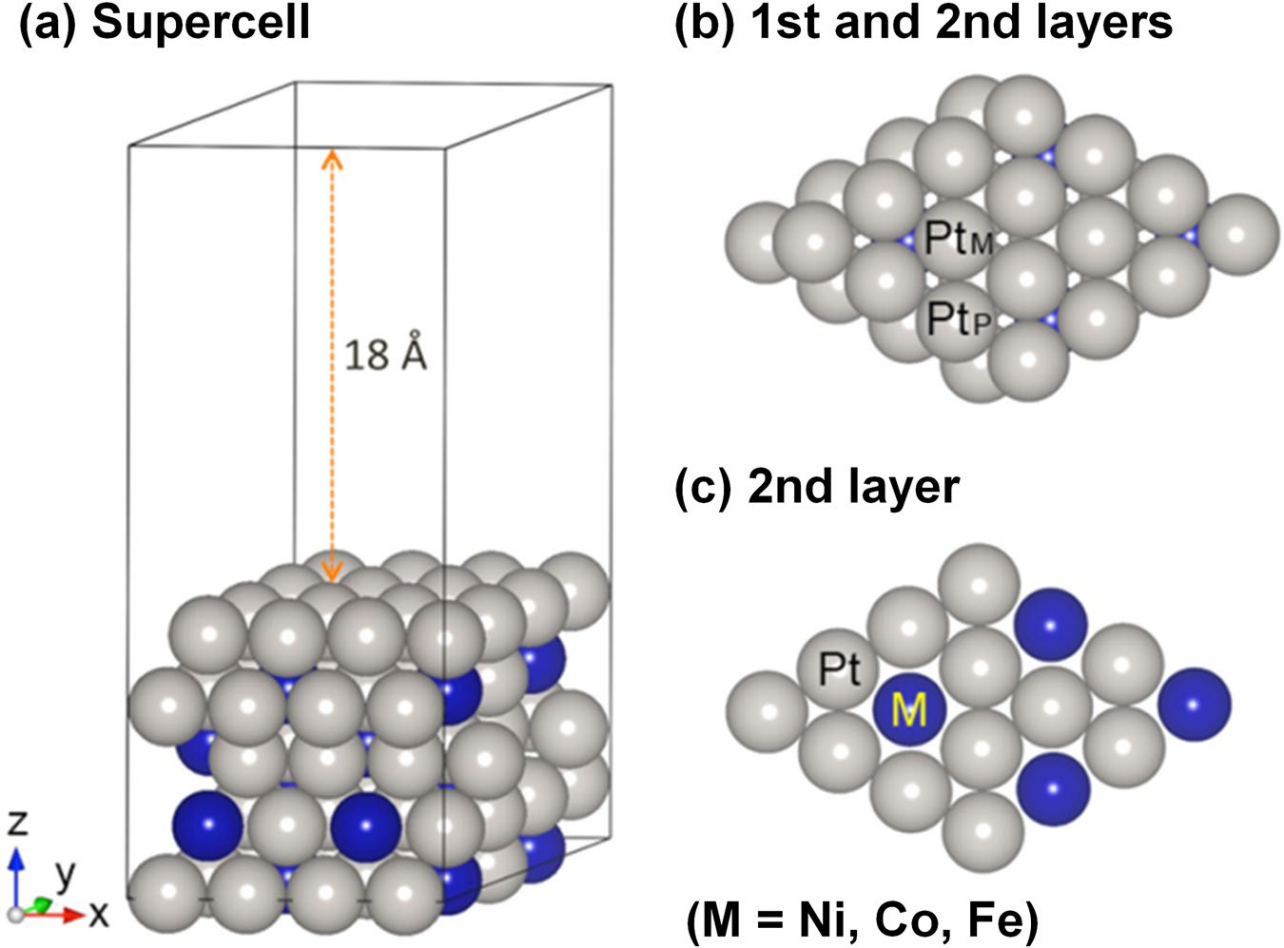
Model structure for the DFT calculation. (**a**) Supercell structure. (**b**) Top view of the supercell showing the first and second layers. (**c**) Top view of the second layer.

To simulate the Pt_skin_/Pt_3_M/C or Pt/C catalyst surfaces using DFT calculations, we prepared Pt_skin_/Pt_3_M surface models (M = Pt, Ni, Co, or Fe), as shown in Fig. 4. The top surface layer was pure Pt atoms (indicated by Pt_skin_) and corresponds to the Pt skin in the Pt_skin_/Pt_3_M/C catalysts. The subsurface layers consisted of four layers of Pt_3_M(111) structure with two fixed bottom layers representing the alloy core of the Pt_skin_/Pt_3_M/C catalysts. To simulate the Pt/C catalyst surfaces, M was replaced with Pt.

**Figure 5:**
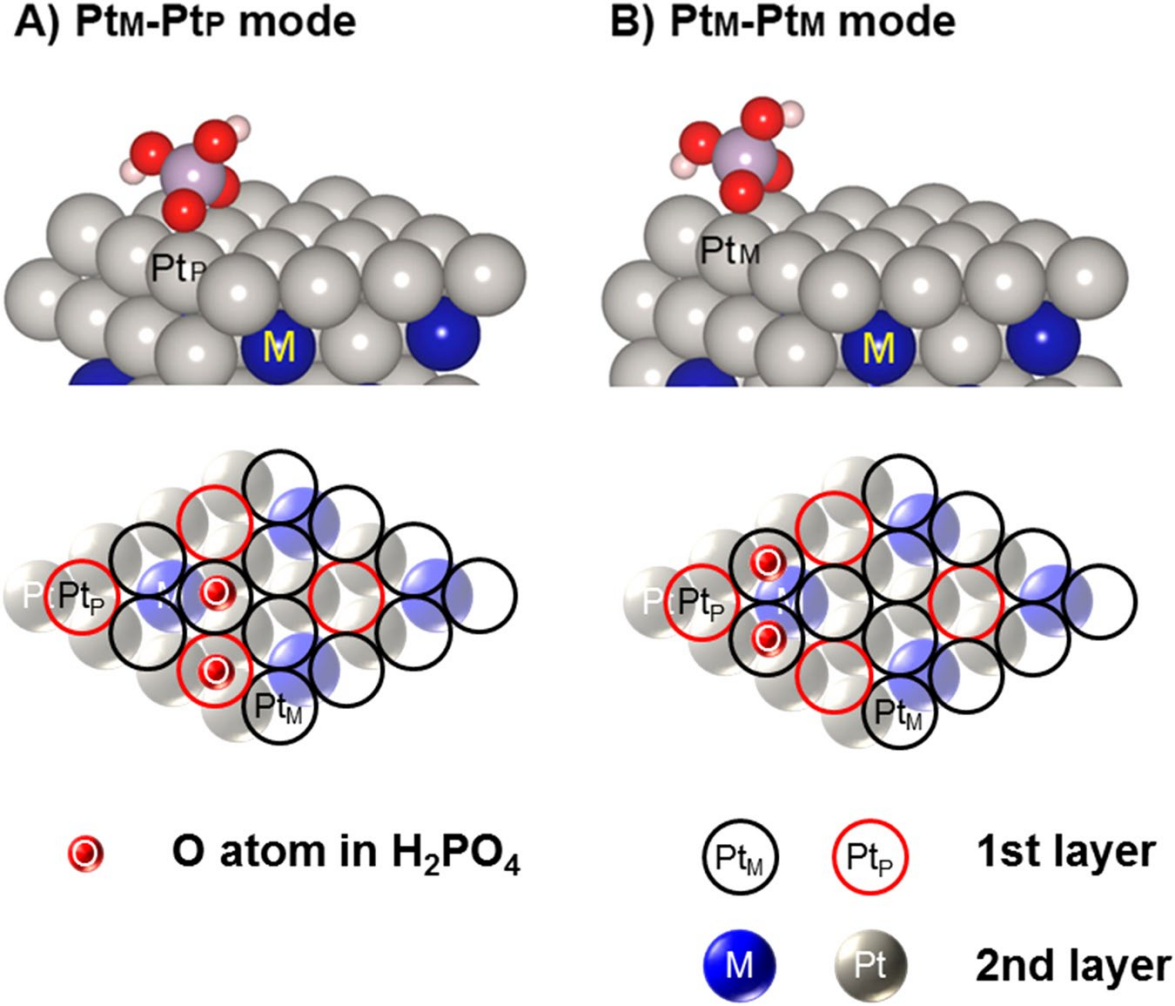
Structure of adsorption sites. (**A**) Pt_M_–Pt_P_ and (**B**) Pt_M_–Pt_M_ modes. Gray, blue, red, purple, and white balls indicate Pt, M (Ni, Co, Fe), O, P, and H, respectively.

Figure 5 shows the adsorption configurations of H_2_PO_4_ on the Pt_skin_/Pt_3_M model surfaces. Two adsorption sites of H_2_PO_4_ on the Pt_skin_/Pt_3_M surfaces were taken into account owing to the M atom location in the first subsurface layer: (1) surface Pt atoms connected to three Pt subsurface atoms (Pt_P_) and (2) surface Pt atoms linked to two Pt atoms and one M atom (Pt_M_). Considering the phosphoric acid adsorption geometry, phosphoric acid adsorption on the Pt_skin_/Pt_3_M surfaces has two adsorption modes: Pt_M_–Pt_M_ and Pt_M_–Pt_P_.

**Figure 6:**
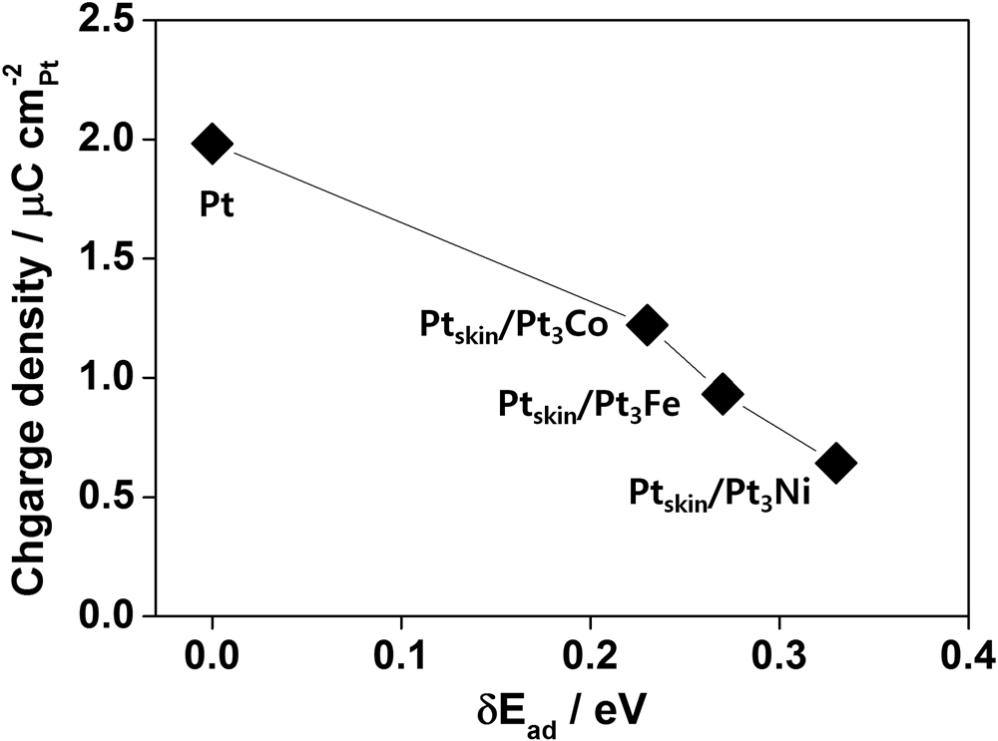
Phosphoric acid adsorption strength. δE_ad_ (differences in ΔE_ad_) determined from the DFT calculation (y axis) as a function of accumulated charge density from 0.35 to 0.45 V in the electrochemical analysis.

To more easily compare the adsorption strength of the phosphoric acid anion on the surfaces, the differences in Δ*E*_ad_ between the Pt(111) and Pt_skin_/Pt_3_M surfaces (δ*E*_ad_) are presented in Fig. 6.

3 $${\rm{\delta }}{E}_{{\rm{ad}}}={\rm{\Delta }}{E}_{\text{ad},\text{Pt3M}}-{\rm{\Delta }}{E}_{\text{ad},\text{Pt}}$$

We find that the comparison of δ*E*_PA_ reveals a more negative δ*E*_PA_ on the Pt_M_–Pt_M_ site, indicating stronger adsorption, than on the Pt_M_–Pt_P_ site (Fig. S6). As positive values of δ*E*_PA_ indicated weaker adsorption, the fact that δ*E*_PA_ on the Pt_M_–Pt_M_ site was lower than that on Pt_M_–Pt_P_ by 0.02 eV (Pt_skin_/Pt_3_Ni) to ~0.07 eV (Pt_skin_/Pt_3_Fe) indicated that PA_ad_ on the Pt_skin_/Pt_3_M prefers the Pt_M_–Pt_M_ site for phosphoric acid adsorption.

From Eq. (3), we see that the adsorption energy on Pt_skin_/Pt_3_M has a higher magnitude than that on the pure Pt surface, indicating weaker adsorption of H_2_PO_4_ on the Pt_skin_/Pt_3_M surface and thus suggesting higher phosphoric acid tolerance. In Fig. 6, the order of δ*E*_PA_ (Pt < Pt_skin_/Pt_3_Co < Pt_skin_/Pt_3_Fe < Pt_skin_/Pt_3_Ni) is the inverse of the order of the accumulated charge density determined from CV (Pt/C > Pt_skin_/Pt_3_Co/C > Pt_skin_/Pt_3_Fe/C > Pt_skin_/Pt_3_Ni/C). As positive values of δ*E*_PA_ indicate weaker adsorption, the inverse relationship between the magnitude of δ*E*_PA_ and the accumulated charge density of phosphoric acid anions (Fig. 3) demonstrates that δ*E*_PA_ predicted well the effect of alloying on the adsorption strength of phosphoric acid despite the simplification of the simulation models. This result supports the observation by He *et al*. that PtNi electrocatalysts show improved phosphoric acid tolerance as compared with Pt^4^, although the ORR activity at 0.85 V reflects that of PtNi electrocatalysts with the maximum phosphoric acid coverage. Experiments under high mass transport conditions must be carried out to evaluate the effect of decreased phosphoric acid adsorption on the ORR activity around 0.6 V. When the thickness of the Pt skin layer was increased, the δE_ad_ of Pt_skin_/Pt_3_Ni/C gradually decreased (0.33 eV (1 Pt layer) → 0.28 eV (2 Pt layers) → 0.24 eV (3 Pt layers), indicating that the electronic effect of sublayers on the surface Pt atoms decreased. A similar decrease was also observed for Pt_skin_/Pt_3_Co/C and Pt_skin_/Pt_3_Fe/C.

**Figure 7:**
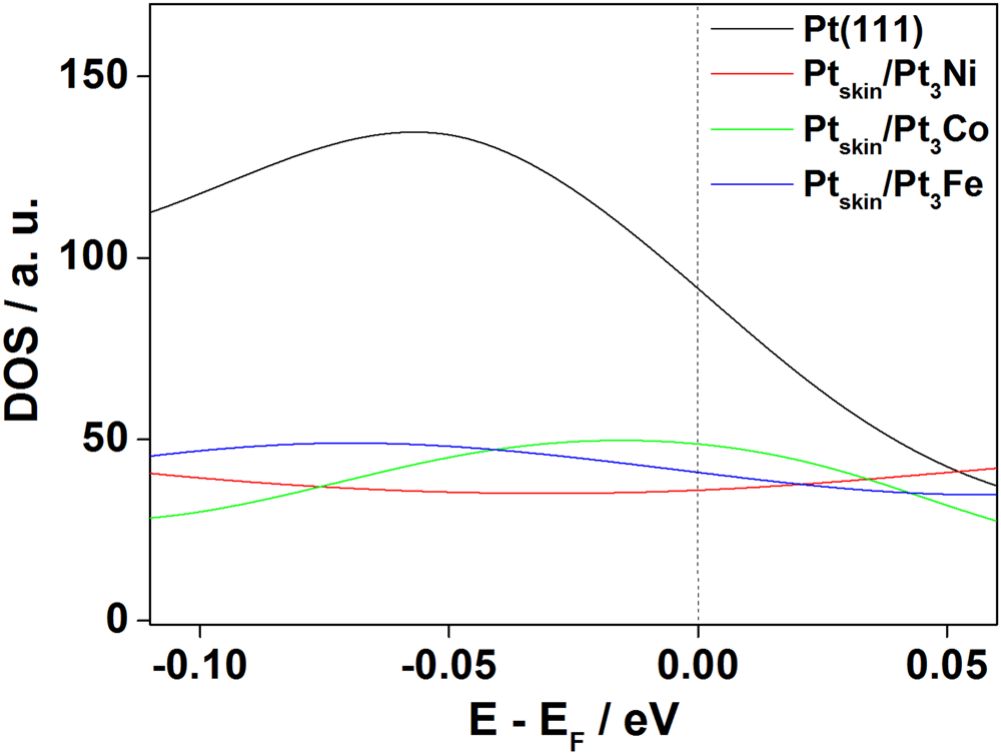
Density of states (DOS) near the Fermi level.

Our DFT calculations revealed that the order of the density of states at the Fermi level (DOS_Ef_) is Pt_3_Ni < Pt_3_Fe < Pt_3_Co < Pt, as shown in Fig. 7, which is the same order as the adsorption strength. As pointed out by Hyman and Medlin^27^, this observation is attributed to the fact that electrons located at or near the Fermi level may participate relatively readily in interactions with adsorbates. As DOS_Ef_ determines the availability of electrons for bonds, the higher DOS_Ef_ value supported stronger adsorption^27^. At the same time, the greater number of unoccupied states immediately above the Fermi level that accompanies the higher DOS_Ef_ also increased the adsorption strength, as the unoccupied density of states decreased the antibonding repulsion of the Pt–O bond^27^. As adsorption of phosphoric acid also forms Pt–O bonds, the increased adsorption strength was predicted from the higher DOS_Ef_ by analogy with the chemical bond formed.

The relative position of the d-band center with respect to the Fermi level (*ε*_d_), which indicates the average energy of the Pt d-bands (Table 1), also predicted the weaker adsorption on the Pt_skin_/Pt_3_M surfaces than on Pt(111) because the d-band center of Pt_skin_/Pt_3_M is closer than that of Pt(111)^27–30^. However, the differences in adsorption strength among the Pt_skin_/Pt_3_M surfaces could not be explained by the position of the d-band centers. This suggests that the average energy of the d-band is insufficient to capture the adsorption strength change due to alloying, and it may be necessary to analyze the individual structures of the d-bands in the Pt_skin_/Pt_3_M surfaces.

**Table 1:** Relative position of d-band centers of 1st layer Pt atoms with respect to the Fermi level.

	Relative position of d-band center (eV)		
	Pt_M_	Pt_P_	Pt_M_ + Pt_P_
Pt_skin_/Pt_3_Ni	−2.653	−2.529	−2.622
Pt_skin_/Pt_3_Co	−2.700	−2.537	−2.659
Pt_skin_/Pt_3_Fe	−2.645	−2.422	−2.589
Pt(111)	−2.414		

## Conclusions

The adsorption strength of phosphoric acid on Pt_skin_/Pt_3_M/C electrocatalyst surfaces was investigated using electrochemical measurements and DFT calculations. Electrochemical analysis demonstrated a decreased adsorption charge density near the onset potential region when Pt was alloyed with Co, Fe, or Ni. DFT calculations predicted weaker adsorption of phosphoric acid with alloying by Co, Fe, or Ni in the subsurface layers. Both the experimental and theoretical results revealed that the Pt alloyed with Ni showed the weakest adsorption of phosphoric acid, followed by the alloys with Fe and Co, and pure Pt. In addition, the decreased ability to adsorb phosphoric acid is attributed to the lower density of states at the Fermi level (DOS_Ef_), suggesting that Pt_skin_/Pt_3_Ni/C has the higher phosphoric acid tolerance.

The current DFT results logically support the experimental observation without consideration of the solvation effect and aid in the fundamental understanding of H_2_PO_4_ adsorption on Pt_3_M. Nonetheless, in future studies on expanding the current DFT and experimental work to other transition metals, the solvent effect must be taken into account for better understanding of related concepts.

## Methods

### Electrochemical analysis

Electrochemical analysis was performed using a conventional three-electrode setup, with a catalyst-coated glassy carbon (GC) electrode, saturated calomel electrode, and Pt wire as the working, reference, and counter electrodes, respectively. To prepare the catalyst layer on the GC electrode, a homogeneous mixture of the electrocatalysts, Nafion solution (Sigma-Aldrich), and 2-propanol (Sigma-Aldrich) was placed on the GC and then dried under a gentle Ar (99.999%) stream. The potential of the working electrode was controlled by a potentiostat (AUTOLAB PGSTAT) and was reported with respect to the normal hydrogen electrode (NHE). Pt_skin_/Pt_3_M/C (M = Ni, Co, Fe) electrocatalysts were prepared using electrochemical leaching of M from pristine Pt_3_M/C (M = Ni, Co, Fe) electrocatalysts (E-TEK). The catalyst-coated working electrode was subjected to potentiodynamic treatment (CV) in Ar-saturated 0.1 M HClO_4_ (0.05–1.00 V, scan rate of 20 mV s^−1^) followed by potential cycling 10 times at −0.2 to 0 V (20 mV s^−1^) to ensure complete reduction of Pt oxides. Then, the working electrode was gently but thoroughly rinsed with deionized water to remove perchlorate ions and dissolved M ions.

The formation of a Pt skin was confirmed using CV and CO-stripping analysis in 0.1 M KOH. The CV curve was recorded in 0.1 M KOH at a scan rate of 20 mV s^−1^ at −0.65 to 0.2 V. To perform CO-stripping voltammetry, the working electrode was held at −0.55 V for 20 min in CO-saturated 0.1 M KOH; then, the electrolyte was purged with Ar for 30 min to remove the CO in the electrolyte. The first anodic sweep in CV analysis afforded electrochemical oxidation of adsorbed CO, resulting in an anodic peak in the CV curve at around 0 V. Electrochemical adsorption of phosphoric acid was analyzed using CV analysis in 0.1 M HClO_4_ with and without 10 mM H_3_PO_4_ at 0.05–0.8 V (20 mV s^−1^).

### Computational methodology

Spin-polarized DFT calculations were performed using the Vienna *ab initio* Simulation Package^31–34^ with the projector-augmented wave^35, 36^ method. Electron exchange-correlation functionals were represented using the generalized gradient approximation and the Perdew and Wang^37^ approximation (PW91). A kinetic energy cutoff of 350 eV was used with a plane-wave basis set. A rhomboidal supercell (10.97 × 10.97 × 26.88 Å^3^) was used to model the Pt_3_M(111)−*p*(4 × 4) structures (where M = Ni, Co, Fe). The Pt_3_M surfaces were represented as a five-layer slab including one Pt skin layer and a 17.8-Å-thick vacuum region to prevent interactions between periodic images. Brillouin zone integration of the Pt_3_M(111)−*p*(4 × 4) surfaces was performed using 2 × 2 × 1 Monkhorst–Pack *k*-point meshes^38^ and first-order Methfessel–Paxton smearing^39^ with a width of 0.05 eV. The bottom two layers were fixed, and the top three layers were fully relaxed. The isolated H_2_PO_4_ was optimized in the same supercell of the Pt_3_M systems, in which Brillouin zone integration was carried out for the gamma-point only. All atoms were fully relaxed and optimized until the forces were reduced below 0.05 eV/Å. Details of the calculation methods are discussed in ESI.

## Electronic supplementary material

Supplementary Information
