# Supplementary material for: Transition metal alloying effect on the phosphoric acid adsorption strength of Pt nanoparticles: an experimental and density functional theory study
Source: Sci Rep. 2017 Aug 3;7:7186. doi: 10.1038/s41598-017-06812-w (PMC5543171; doi:10.1038/s41598-017-06812-w)
Supplement: Supplementary file 1 — Supplementary Information [file 41598_2017_6812_MOESM1_ESM.doc]

Transition metal alloying effect on the phosphoric acid adsorption strength of Pt nanoparticles: an experimental and density functional theory study

Hee-Young Park,^1,†^ Dong-Hee Lim,^2^,† Sung Jong Yoo,^1^ Hyoung-Juhn Kim,^1^

Dirk Henkensmeier,^1^ Jin Young Kim,^1,3^ Hyung Chul Ham,^1^,* Jong Hyun Jang^1,3^,*

^1^ Fuel Cell Research Center, Korea Institute of Science and Technology, Hwarangno 14-gil 5, Seongbuk-gu, Seoul 02792, Republic of Korea

^2^Department of Environmental Engineering, Chungbuk National University, Cheongju Chungbuk 28644, South Korea

^3^Green School, Korea University, Seoul 02841, Republic of Korea

*Corresponding Author

Tel.: +82-2-958-5287; Fax: +82-2-958-5199; E-mail: jhjang@kist.re.kr

*Corresponding Author

Tel.: +82-2-958-5889; Fax: +82-2-958-5199; E-mail: hchahm@kist.re.kr

^†^These authors contributed equally to this work.

**Figure S1.** Cyclic voltammogram of Pt/C (black), Pt_skin_/Pt_3_Ni/C (red), Pt_skin_/Pt_3_Co/C (green), and Pt_skin_/Pt_3_Fe/C (blue) in 0.1 M KOH. Electrochemical surface area of Pt of the electrocatalysts were determined using the underpotentially deposited hydrogen charge (H_upd_) measured in 0.1 M HClO_4_ and charge density constant (0.210 mC cm_Pt_^-2^).


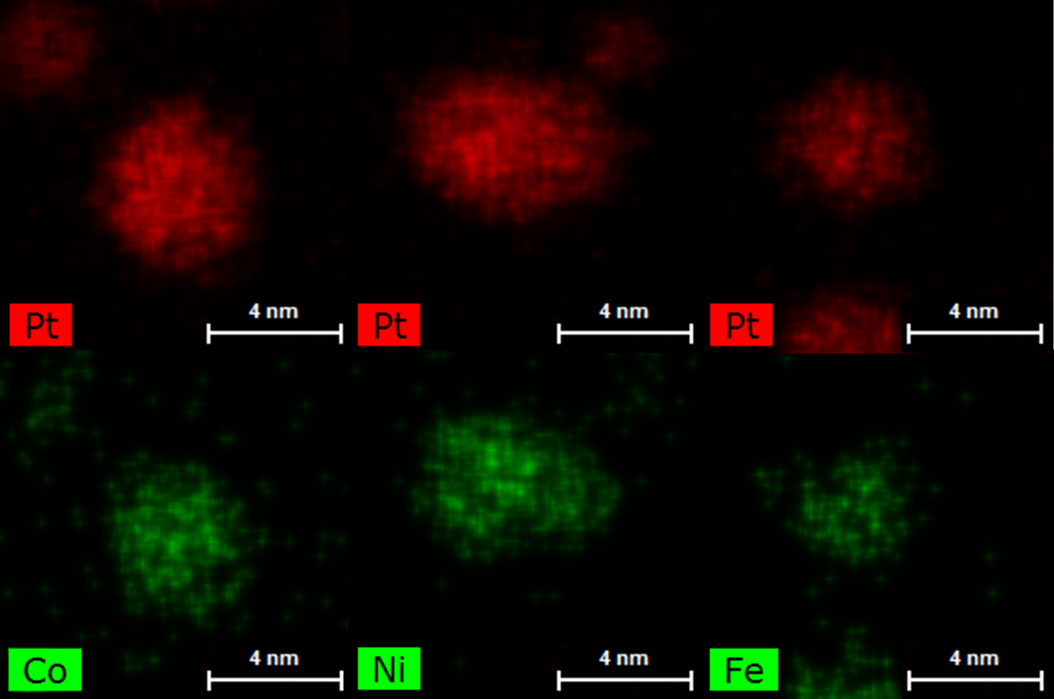


**Figure S2.** Elemental mapping image of Pt in (a) Pt_skin_/Pt_3_Co/C, (b) Pt_skin_/Pt_3_Ni/C, and (c) Pt_skin_/Pt_3_Fe/C. Elemental mapping image of (d) Co, (e) Ni, and (f) Fe in Pt_skin_/Pt_3_M/C.


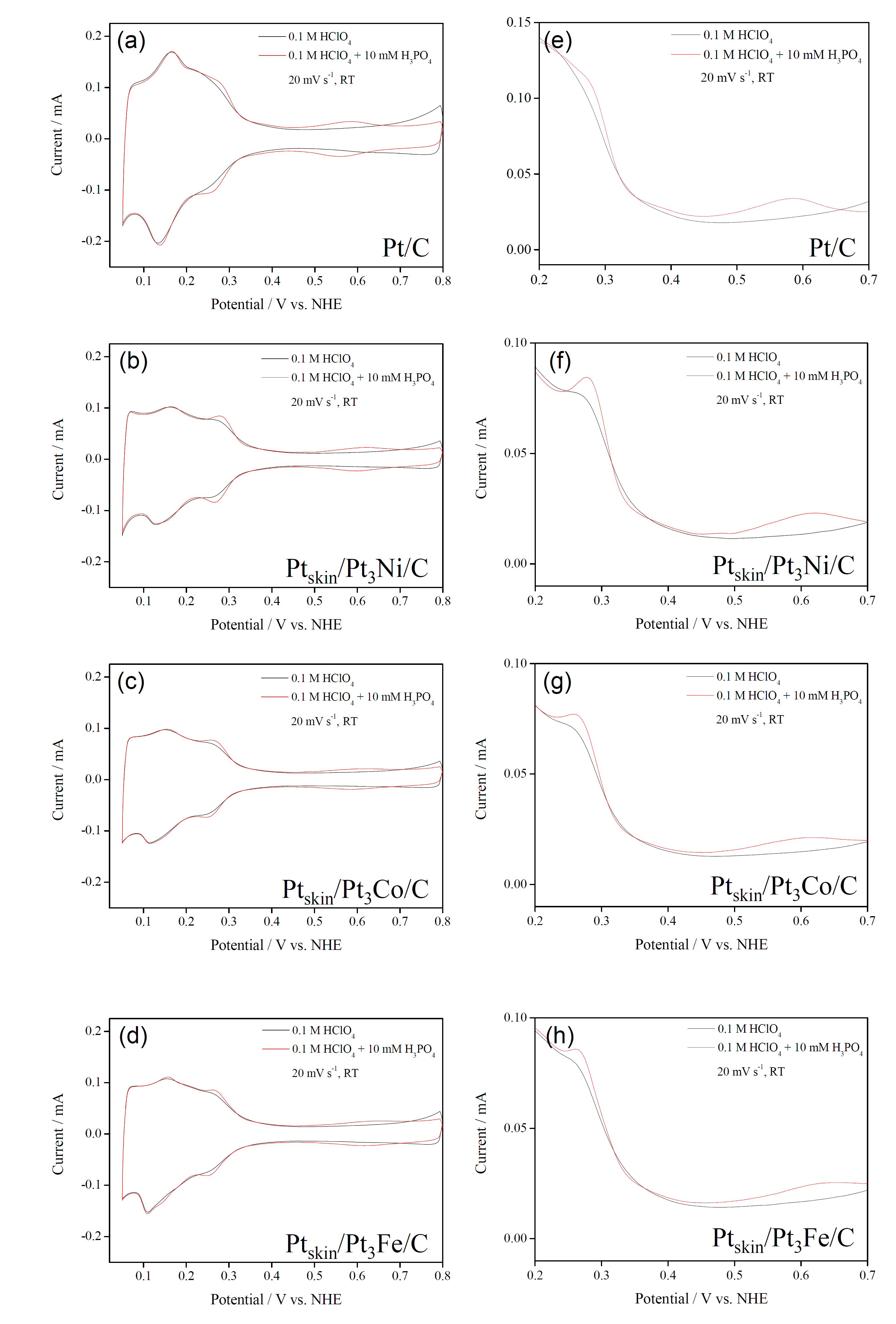


**Figure S3.** Cyclic voltammogram of (a, e) Pt/C, (b, f) Pt_skin_/Pt_3_Ni/C, (c, g) Pt_skin_/Pt_3_Co/C, and (d, h) Pt_skin_/Pt_3_Fe/C in 0.1 M HClO_4_ (black) and 0.1 M HClO_4_ + 10 mM H_3_PO_4_ (red).


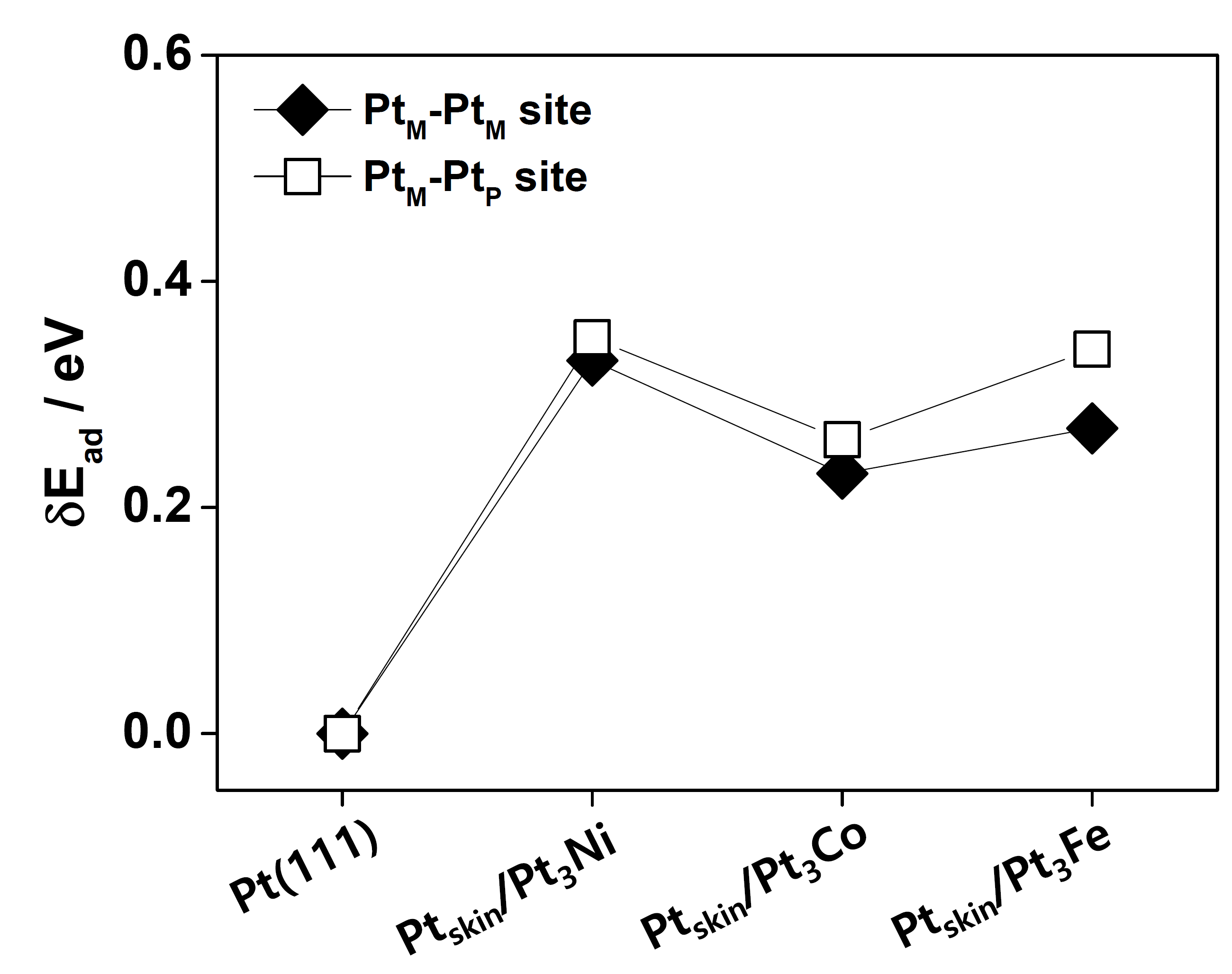


**Figure S4.** Differences in Δ*E*_ad_ between the Pt(111) and Pt_skin_/Pt_3_M surfaces (δ*E*_ad_) on Pt(111) and Pt_skin_/Pt_3_M(111) surfaces.

**Details of DFT calculations**

**(1) Multi-layer Pt skins**

Two and three Pt skin layers were made based on the single Pt skin layer by stacking one and two more Pt skin layers, respectively. The adsorption energies of H_2_PO_4_ on the multi-layer Pt skin Pt_3_M were calculated using the same computational method as described in Eq.(2) of the paper.

**(2) Dipole moment correction**

The effect of dipole correction was tested by adding the dipole moment correction options provided by VASP as follows.

LDIPOL = .TRUE.

IDIPOL = 3

Although the dipole correction may change the total energies of Pt_3_M with H_2_PO_4_, clean Pt_3_M, and gas-phase H_2_PO_4_, the difference in the H_2_PO_4_adsorption energies with and without the dipole correction was negligible (~ 2 meV) due to the fact that the adsorption energy is relative physical property between adsorbed species and separated species.

**(3) True energy minimum of optimized structures**

It is important to check if there are any imaginary frequencies to ensure that the optimized structures corresponded to a true energy minimum. To confirm this, we have conducted the frequency analyses of the optimized structure of Pt_3_Ni with H_2_PO_4_ and verified that there was no imaginary frequency.

**(4) Solvation effect**

Taking into account direct or indirect solvent effect may be limited in DFT calculations due to impractical use of computational resources. According to our previous study^[1]^ examining the adsorption of chloroethene on Fe(110), the solvation effect led to increasing the adsorption energy difference without changing the trend of adsorption strength compared to those without solvation effect. Thus it may be assumed that solvent effect would increase the adsorption strength difference of H_2_PO_4_ on Pt_3_M and provide more accurate information; however, without the solvent effect calculations the current DFT results logically supported the experimental observation and helped the fundamental understanding on the H_2_PO_4_ adsorption by analyzing the DOS results.

Furthermore, solvation effect may be dominantly affected by the structures of adsorbates rather than the catalyst surfaces. For example, Peterson et al.^[2]^ took into account the effect of solvation for the CO_2_ reduction intermediates on the Cu(211) surface to analyze the CH_4_ production pathways. In the DFT calculations, they assumed that due to solvation the adsorbates of OH*, R*-OH, CO*, and CHO* were stabilized by approximately 0.5, 0.25, 0.1, and 0.1 eV, respectively, in which the solvation energy were obtained from different types of metal surfaces. Thus, in the case of the current study where the same adsorbate of H_2_PO_4_ was analyzed in the different Pt_3_M systems, it may be assumed that solvation effect may affect the magnitude of the adsorption strength, but may not change the trend of the adsorption strength.

**References**

[1]: Lim, D.-H., Lastoskie, C. M., Soon, A. & Becker, U., *Environmental Science & Technology* 43, 1192-1198 (2009).

[2]: Peterson, A. A., Abild-Pedersen, F., Studt, F., Rossmeisl, J., Nørskov, J. K., *Energy & Environmental Science* 3, 1311-1315 (2010), [Supplementary Information page 3].

[3]: Savizi, I. S. P., Janik, M., *Electrochimica Acta* 56, 3996-4006 (2011).
